# Assessment of Common and Emerging Bioinformatics Pipelines for Targeted Metagenomics

**DOI:** 10.1371/journal.pone.0169563

**Published:** 2017-01-04

**Authors:** Léa Siegwald, Hélène Touzet, Yves Lemoine, David Hot, Christophe Audebert, Ségolène Caboche

**Affiliations:** 1 Gènes Diffusion, Douai, France; 2 CRIStAL (UMR CNRS 9189 University of Lille, Centre de Recherche en Informatique, Signal et Automatique de Lille) and Inria, Villeneuve d'Ascq, France; 3 Univ. Lille, CNRS, Inserm, CHU Lille, Institut Pasteur de Lille, U1019 - UMR 8204 - CIIL - Centre d'Infection et d'Immunité de Lille, Lille, France; 4 PEGASE-Biosciences, Institut Pasteur de Lille, Lille, France; Massey University, NEW ZEALAND

## Abstract

Targeted metagenomics, also known as metagenetics, is a high-throughput sequencing application focusing on a nucleotide target in a microbiome to describe its taxonomic content. A wide range of bioinformatics pipelines are available to analyze sequencing outputs, and the choice of an appropriate tool is crucial and not trivial. No standard evaluation method exists for estimating the accuracy of a pipeline for targeted metagenomics analyses. This article proposes an evaluation protocol containing real and simulated targeted metagenomics datasets, and adequate metrics allowing us to study the impact of different variables on the biological interpretation of results. This protocol was used to compare six different bioinformatics pipelines in the basic user context: Three common ones (mothur, QIIME and BMP) based on a clustering-first approach and three emerging ones (Kraken, CLARK and One Codex) using an assignment-first approach. This study surprisingly reveals that the effect of sequencing errors has a bigger impact on the results that choosing different amplified regions. Moreover, increasing sequencing throughput increases richness overestimation, even more so for microbiota of high complexity. Finally, the choice of the reference database has a bigger impact on richness estimation for clustering-first pipelines, and on correct taxa identification for assignment-first pipelines. Using emerging assignment-first pipelines is a valid approach for targeted metagenomics analyses, with a quality of results comparable to popular clustering-first pipelines, even with an error-prone sequencing technology like Ion Torrent. However, those pipelines are highly sensitive to the quality of databases and their annotations, which makes clustering-first pipelines still the only reliable approach for studying microbiomes that are not well described.

## Introduction

Metagenomics based on high-throughput sequencing (HTS) helps biologists unveil a large part of the constitutive microorganisms of a microbiota. This culture-free application has spread widely into microbiology studies over the past decade [1], and is now applied to several domains such as clinical microbiology to obtain a microbiota signature associated with a clinical picture [2], industrial processes for quality control [3], or environmental communities studies [4,5]. The term metagenomics can refer to two distinct methods: shotgun metagenomics and targeted metagenomics.

Shotgun metagenomics usually considers the entire genomic content of a sample, by extracting and sequencing the total DNA. As a result, this comprehensive approach offers a rich picture of a microbiota, and provides the opportunity to simultaneously explore the taxonomic and functional diversity of microbial communities [6]. However, shotgun metagenomics is still very expensive and the data analysis is a challenging task, due both to the size and the complex structure of the data [7]. This is a significant obstacle to common applications.

Targeted metagenomics, also named *metagenetics* [8] or amplicon-based metagenomics, focuses on a taxonomically informative genomic marker only. This discriminating locus is amplified prior to sequencing, greatly reducing the amount of data to be sequenced and analyzed. For prokaryotic studies, which is the focus of this article, the target of choice is a portion of the 16S rDNA gene, composed of both conserved and hypervariable regions specific to different prokaryotic taxa. Targeted metagenomics can nowadays be integrated into routine processes. Indeed, the advent of benchtop sequencing allows HTS studies at a smaller scale and lower price. It has made targeted metagenomics accessible to common laboratories, hospitals and industries [9,10]. Roche was the first company to introduce benchtop sequencing in 2010 with the release of the 454 GS Junior pyrosequencer, announced to be discontinued in 2016. Recent benchtop solutions are the Life Technologies Ion Torrent sequencers, using semiconductor ion detection, and the Illumina MiSeq and NextSeq sequencers, using fluorescent dye detection. One major difference between these sequencing technologies is the type and abundance of sequencing errors [11] impacting the base-calling quality.

Once the raw sequencing reads have been produced from the amplified targeted 16S rDNA region of a metagenome, the next step is to analyze them to estimate the microbial diversity and taxa composition. To this end, an increasing number of bioinformatics analysis pipelines are available [12]. They are specifically designed to link several steps together, such as read preprocessing, chimera detection, Operational Taxonomic Unit (OTU) clustering, or taxonomic assignment [13]. These pipelines integrate many algorithms to offer the widest range of possibilities. As a consequence, they require advanced bioinformatics skills and computing resources, and can discourage users not familiar with the diversity of existing analytical processes. Each pipeline proposes its own guidelines, with configuration profiles, reference databases and recommended analytical steps. Choosing a pipeline with a set of parameters and algorithms for a given application can quickly become a difficult task without an evaluation protocol.

Some efforts have recently been made to assess and evaluate the principal existing targeted metagenomics bioinformatics processes. However, these comparative studies mostly focus on a single analytical step, such as the impact of sequence preprocessing [14], OTU clustering [15] or taxonomic assignment [16,17]. They use datasets (real or simulated) from the Illumina [18] and 454 sequencing platforms only. A more global work [19] compared the results of two targeted metagenomics pipelines on real human gut 454 metagenomic datasets for which the constituent organisms and their abundances are unknown: the accuracy of results cannot therefore be assessed objectively. Another study [7] evaluates the performance of different analytical pipelines on artificial controlled datasets, but the latter were simulations of whole genome shotgun metagenomic reads, and not targeted amplicon sequencing outputs. To our knowledge, no study has ever compared global targeted metagenomics analysis methods in their entirety: the latter being intended for non-expert users, and defined by a pipeline and its usage guidelines (including advised parameters and database).

**Fig 1:**
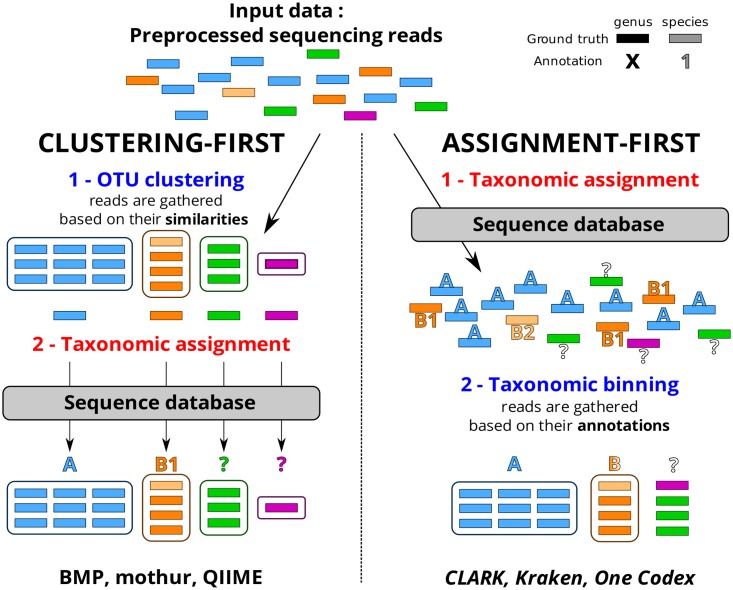
Distinctions between clustering-first and assignment-first approaches. A question mark indicates an unclassified read and/or taxon.

An extensive literature survey allowed the identification of bioinformatics pipelines developed for targeted metagenomics and WGS metagenomics analyses, aiming to estimate the taxonomic composition and diversity of samples. We characterize two distinct methodologies that we name clustering-first and assignment-first (Fig 1). Clustering-first approaches, also called alignment-based approaches, are almost exclusively the most represented for targeted metagenomics analyses. They start with an OTU-clustering step where reads are gathered into OTUs based on their sequence similarities. From each cluster, a representative sequence is extracted according to different methods (consensus, longest or more abundant sequence, etc.). This sequence is then aligned to each of the 16S rDNA sequences of a reference database using a homology search tool. The representative sequence and the OTU it belongs to are finally assigned to a taxonomic group by inspecting the best alignments.

Assignment-first approaches have emerged more recently for metagenomic analyses. These methods do not group the reads based on their intrinsic similarities, but first compare all of them to a database of reference (e. g. by k-mer discrimination [20,21] or read mapping [22]). They assign the lowest taxonomy possible to each read based on those comparisons, or a lower common ancestor (LCA) for a group of sequences of the same taxonomy within the reference database. Afterwards, the reads are grouped into different taxonomic units based on their annotations.

These two analytical approaches, clustering-first and assignment-first, are conceptually different and can lead to different results. For example, in Fig 1, unlike assignment-first approaches, clustering-first approaches allow the discrimination of unclassified reads (shown in green and purple). However, they mislabel the OTU (in light orange) as species B1 due to the reference sequence selected for this taxon, whereas assignment-first approaches acknowledge a possible mix of the species B1 and B2 in the B genus.

In this paper, we introduce a complete evaluation protocol to compare targeted metagenomics pipelines in their entirety and observe how the analytical process can change the biological interpretation. In order to study the impact of different variables (e.g. sequencing throughput, sequencing error rate…), we decided to mainly use simulated datasets. Indeed, simulated data represents very controlled conditions discarding some experimental biases such as PCR biases and chimera, contrary to the use of mock bacterial communities, which are too expensive to create and sequence in such a diversity of contexts. We simulated sequencing reads from bacterial genomes with different proportions representative of different metagenomic contexts. To cross-validate our observations with simulated datasets, we also included real targeted metagenomics data [23]. To evaluate the analysis of those datasets by any pipeline, this evaluation protocol also includes universal comparison metrics such as clustering and diversity indexes, as well as a variety of comparison criteria such as the choice of the16S rRNA domain, robustness to sequencing errors, computational requirements, etc.

In this study, we decided to focus on 6 pipelines described in Table 1: 3 based on the clustering-first approach, and 3 based on the assignment-first approach. Mothur [24] and QIIME [25] are 2 clustering-first command-line packages. BMP [26] proposes guidelines specific to an Ion Torrent context, using QIIME and UCLUST with parameters fitted to this technology. One Codex [27] is an assignment-first web service, and Kraken [20] and CLARK [21] are assignment-first command-line tools. In order to be in a non-expert user context, all the pipelines were run with their default settings, following each pipeline guidelines. The aim of this study is i) to test the use of assignment-first pipelines in the targeted metagenomics context, which was never done before to our knowledge and ii) to study the impact of analysis steps on the biological interpretation in the basic user context.

**Table 1:** Description of the 6 pipelines compared in this study.

	Clustering-first			Assignment-first		
	mothur	BMP	QIIME	Kraken	CLARK	One Codex
**Version**	1.35.1	Dez. 2014	1.9.0	0.10.5-beta	1.1.2	open beta
**Default database**	SILVA 119	Greengenes 13.8	Greengenes 13.8	MiniKraken 20141208	RefSeq 71 adaptation	OneCodex 28k (proprietary)
**Alternative databases**	Greengenes 13.8	SILVA 119	SILVA 119	NA	NA	RefSeq 65 SILVA 119
**Interface**	Local command-line					Web server
**Reference**	[24]	[26]	[25]	[20]	[21]	[27]

## Results

### An evaluation protocol helps to estimate how far analytical results are from ground truth

As shown in previous studies [14–19], analysis pipelines produce a wide heterogeneity of results and give a somewhat different picture of the composition of a given sample. It points out the need for a comparison protocol with fitted metrics and datasets to determine the origin of such differences. Real data is unbiased but difficult to interpret because of the lack of knowledge of the nature and proportions of the organisms really present in the samples.

In 2007, the FAMeS (Fidelity of Analysis of Metagenomic Samples) datasets were published [28] in order to standardize comparisons between tools assembling and annotating metagenomes. They described three artificial bacterial metagenomic compositions: Low Complexity (LC) with a dominant species, Medium Complexity (MC) with few dominant species, and High Complexity (HC) with species equally distributed. These three levels of complexity typically correspond to bioreactor communities, acid mine drainage biofilm, and agricultural soils respectively. FAMeS datasets have been used in several studies [29–31] to simulate whole shotgun metagenomic datasets. In this study, the FAMeS metagenomes compositions (51 families and 69 genera) were adapted to targeted metagenomics sequencing and integrated into a pipeline evaluation protocol.

**Fig 2:**
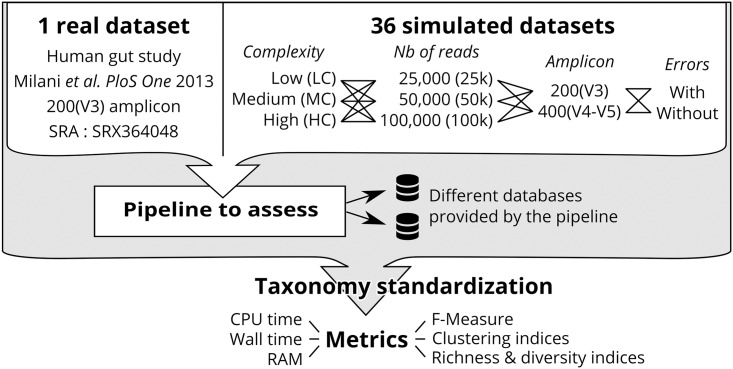
Schematic overview of the evaluation protocol.

We introduced several parameters that allow the consideration of the variability inherent in the design of different sequencing experiments. The first parameter is the choice of primers and therefore the 16S rDNA sequence region to be amplified. Here, we selected two domains: V2 (~200nt) and V4-V5 (~400nt). The second parameter is the sequencing throughput, whose impact was studied at three scales: 25k, 50k and 100k reads. The last variable is the addition of a sequencing error model related to the choice of the sequencing technology. The first model is constituted of error-free amplicons, and the second model is constituted of error-prone reads. Error-prone reads were simulated with an Ion Torrent error and size model, which is representative for sequencing technologies with a relatively high error rate. We decided to use raw simulated reads without any filtering or denoising procedure in order to observe the impact of sequencing errors in targeted metagenomics pipelines. This gives a total of 36 simulated datasets, which are schematically represented in Fig 2 (see Material and Methods for more details). A real human gut Ion Torrent sequencing dataset [23] was also integrated into the evaluation protocol, in order to confirm the observations made on simulated datasets.

All 6 pipelines described in the background were run on these 37 datasets. They were executed with their respective recommended database, but also with alternative databases to observe the impact of this change on the results. To estimate the quality of the taxonomic assignment obtained, our evaluation protocol includes the computation of a series of complementary metrics. The first one is the F-measure, which is the harmonic mean of precision and recall, and allows the comparison of the quality of results between pipelines. We also use richness, diversity and clustering indexes to assess proper reads distribution into different taxonomic units and diversity estimation. This evaluation is performed both at the family (abbreviated F) and genus (abbreviated G) taxonomic levels. Finally, we also measure computational requirements (memory, CPU and running time).

To be able to compare the results on a common ground while using different databases and taxonomies, we chose to normalize the taxonomic assignments by using the NCBI Taxonomy. All reads' assignments were converted in the NCBI Taxonomy at their lowest taxonomic rank, and matching parent taxonomic ranks at the family and genus level.

In the next sections, we chose to display representative results highlighting different pipelines' behaviors (all results available in S1 File).

### Choosing a different amplified region does not systematically change the results significantly

**Fig 3:**
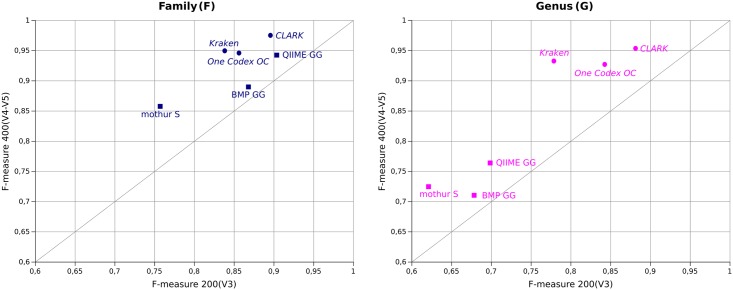
Comparison of F-measures between the 200(V3) and 400(V4-V5) amplicon at the family level (left) and at the genus level (right) on the HC 50k dataset with error simulation.

The choice of primers and therefore the 16S rDNA sequence region to be amplified is a well-known source of bias and differences in results for targeted metagenomics studies [32]. High complexity (HC) datasets simulated on the 200(V3) and 400(V4-V5) amplicons allow the evaluation of the pipelines' behaviors on both amplicons with no composition bias, since all taxa are equally represented. Note that the change of amplicon size cannot be dissociated from the change of informational content, since both are intrinsically related. Fig 3 highlights the impact of amplicon change on the F-measure for each pipeline on the 50k simulated Ion Torrent reads. The F-measure (the closer to 1 the better) takes precision and recall into account. For all pipelines, precision is always higher than recall, meaning that all pipelines favor specificity over sensitivity.

The change of taxonomic resolution (family to genus) mostly impacts the quality of results of clustering-first pipelines, causing a drop in the F-measure between 10 and 20% induced by a loss of both precision and recall. Assignment-first pipelines are more stable between both resolutions, especially on the 400(V4-V5) amplicon.

Surprisingly, the change of amplicon does not improve the results significantly (in Fig 3, all pipelines are relatively close to the diagonal). Mothur, Kraken, CLARK and One Codex are more impacted, with an F-measure increased by around 10% at the family level when using the 400(V4-V5) amplicon. This is caused by an increase in recall (recall F is increased by 15%, 18.2% and 11% for mothur, Kraken and CLARK respectively) except for One Codex, for which the F-measure increase is caused by both an increase in precision (F 9.6% gain) and recall (F 8.5% gain). QIIME is not significantly affected by the amplicon change, with an F-measure increase at the family level below 4%, which is mainly caused by the proper identification of the two additional families amplified by the 400(V4-V5) primers couple only. BMP is the least impacted by this amplicon change, probably because of its first step of read trimming, removing 25% of the ending nucleotides which means a loss of some information added by the longer 400(V4-V5) amplicon.

With error-free amplicons (S1 File), the improvement in the results using the 400(V4-V5) amplicon is much more significant than on reads with error simulation. This is due to the Ion Torrent error generation, the rate of which increases at the end of reads, distorting the supposedly more discriminating bases in the 400(V4-V5). It is therefore important to evaluate the robustness of each pipeline when encountering sequencing errors.

### Because of sequencing errors, high F-measures do not imply a correct richness estimation

Some high-throughput sequencers generate reads with a significant error rate that could lead to taxon misidentifications. To evaluate this phenomenon, a comparison of the pipelines' performances on datasets with and without errors has been performed.

**Fig 4:**
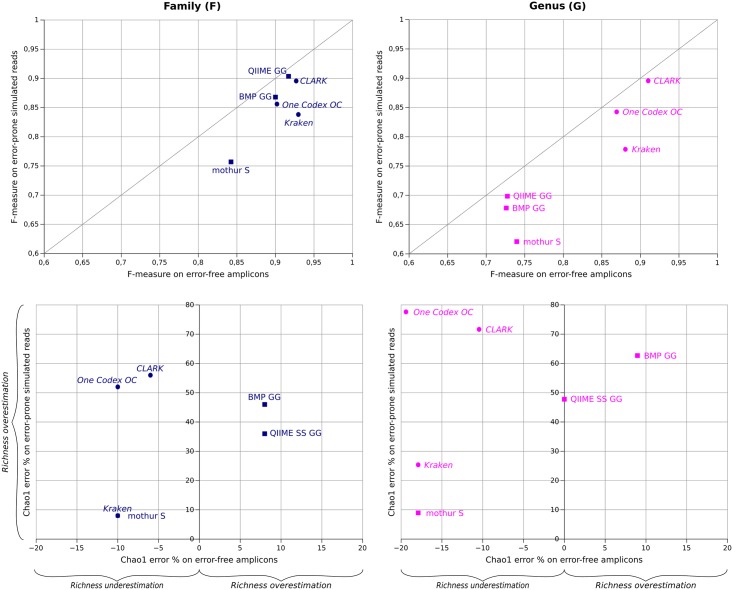
Comparison of F-measures (top) and richness error (bottom) in the error-free and error-prone sequencing models on the 200(V3) HC 50k dataset.

Fig 4 (top) plots F-measures of the HC 200(V3) 50k dataset with and without sequencing error simulation, at the family and genus levels. As observed before, all pipelines generate a higher F-measure at the family resolution and, as expected, the F-measure drops when reads contain sequencing errors. With and without errors, all pipelines present acceptable F-measures (F-measure >0.75) at the family level. Only assignment-first pipelines stay within this F-measure range at the genus level.

When errors are added, most pipelines are affected by a drop in recall (up to 15.6% G for mothur), meaning that a read with errors is considered to be unclassified rather than assigned to the wrong taxon. Mothur and Kraken are the pipelines the most affected by the presence of errors, with an F-measure drop of 8.5% and 9.1% respectively at the family level. QIIME is the pipeline least sensitive to sequencing errors, with an F-measure drop below 3% at both taxonomic levels, mainly due to a drop of recall. Except for One Codex, the precision remains generally stable (<2.5% decrease F and G), and sometimes even increases (~1.5 to 2% F for mothur and QIIME). In that case, adding errors into initial false positive reads that are already on the edge of a classification threshold can tip them over into the unclassified category, explaining the better precision. One Codex is the only pipeline with increasing false positives when adding sequencing errors, with a drop of precision around 12–13% F and G. This does not impact the F-measure too much, since this drop consists of an increase in recall, identified reads (false negatives) becoming wrongly identified (false positives) when errors are added. CLARK shows a moderate drop in precision of around 6% at the family level, which is however reduced to 2% at the genus level, demonstrating its bigger stability at this finer taxonomic resolution.

Sequencing errors also affect the richness estimation (Fig 4, bottom). Richness indexes were computed after taxonomic assignment (all OTUs matching the same taxon were merged and counted as one entity) to be comparable between clustering-first and assignment-first pipelines. Without errors, QIIME and BMP are the only pipelines that overestimate richness, between 8% and 10% Chao1 error at the family level, whereas mothur, Kraken and One Codex all underestimate richness in the same proportions (-10% F Chao1 error). Interestingly, QIIME gets extremely close to ground truth at the genus level (close to 0 on the X-axis), and is the only pipeline to better estimate richness at the genus level than the family level, because the richness overestimation is compensated by the drop in resolution at the genus level. When errors are introduced, all pipelines overestimate richness. Indeed, the sequencing errors imply the formation of a greater number of small OTUs affecting the Chao1 index estimation. In that case, Kraken and mothur are the pipelines the least affected at both the family and genus levels (around the same Chao1 error percentage as without errors) followed by QIIME (36% F Chao1 error percentage increase) and BMP (46% F Chao1 error percentage increase). One Codex and CLARK are the most sensitive to errors in terms of richness, reaching around 50–55% F and 70–80% G Chao1 error percentage increase with errors. This huge variation is explained by the misidentification of many small taxa (<5 reads per taxon) for those pipelines when errors are added. This ranking is the same at the genus level, with an increase in the Chao1 error percentage from family to genus (1% for Kraken, up to 25% increase for One Codex).

As expected, the presence of sequencing errors into targeted metagenomics reads causes both a drop in the F-measure and richness overestimation, but not in the same proportions depending on the pipeline. These results are confirmed by the 25k and 100k datasets (S1 File).

### The richness estimation is affected by variations in the sequencing throughput

Ion Torrent benchtop sequencers allow sample multiplexing: for example, up to 96 samples can be multiplexed on a 318^™^ chip on the Ion Torrent PGM, for a 40k theoretical read throughput per sample. In reality, this amount can vary tenfold from one sample to another. The impact of varying sequencing throughput (25k, 50k and 100k reads) was compared between the pipelines on simulated datasets.

**Fig 5:**
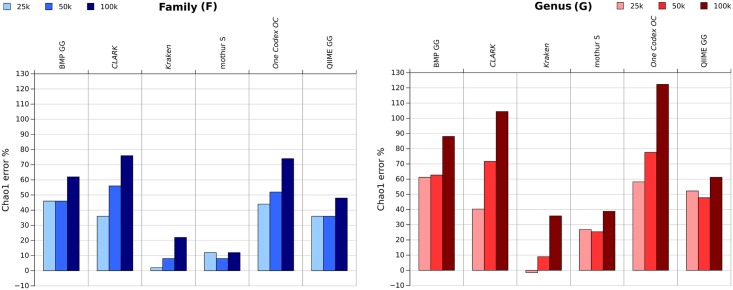
Comparison of the Chao1 error percentage on the 200(V3) HC dataset with sequencing errors simulation considering 25k, 50k and 100k reads, at the family and genus level, after taxonomic merging.

We first tested the effect of throughput variation on error-free datasets. In this context, the richness and F-measure stays stable for all throughputs (S1 File). We then ran the same experiment on error-prone datasets. Fig 5 displays the Chao1 error percentages on the 200(V3) HC dataset for the three different throughputs, 25k, 50k and 100k.

All pipelines overestimate the richness, but two distinct behaviors can be observed. Assignment-first pipelines (Kraken, One Codex and CLARK) show a Chao1 error percentage increase whenever the throughput is also increased. They are the most impacted by sequencing throughput variation, with a Chao1 error percentage of 20% for the 25k dataset, 30% for the 50k one and 40% for the 100k dataset, and even more at the genus level. One Codex and CLARK are the most sensitive to throughput increase, with more than 100% G Chao1 error on the 100k dataset. The assignment step of those pipelines is highly dependent on the number of erroneous k-mers, which increases with higher throughputs. The richness is less overestimated by Kraken, probably because of the reduced k-mer database it was executed with.

Clustering-first pipelines (BMP, QIIME and mothur) are more stable between the 25k and 50k datasets. QIIME and BMP only overestimate the richness for the 100k dataset. QIIME performs better than BMP at both the family and genus levels. Mothur is the pipeline the least sensitive to a change in throughputs, with only an 11% Chao1 error increase at the genus level between 25k and 100k, and almost no variation at the family level.

Clustering-first pipelines are less sensitive to throughput variation, but only after taxonomic merging. The richness and diversity indexes are usually computed before taxonomic merging for those pipelines, and are displayed in Table 2. Here, those metrics were estimated on OTUs formed after read clustering at a threshold of 3% for all pipelines. The richness and diversity overestimation for all pipelines is more significant before taxonomic merging, between a tenfold and a hundredfold. The Simpson Index varies below 10% between the smallest and largest dataset, except for BMP. On the contrary, the Chao1 index is widely impacted by larger datasets, increasing at least 80% of the richness estimation between the 25k and 100k datasets. BMP is the pipeline most affected in terms of richness (199% Chao1 increase between the 25k and the 100k dataset) and diversity (25% Inverse Simpson index increase). Mothur's estimation of richness is also affected by throughput (234% Chao1 increase), but its diversity variation is below 10%. Table 2 also confirms that QIIME SS is an improvement over QIIME U, with the smallest richness variation (80% Chao1 increase between the 25k and the 100k dataset) and below 1% Inverse Simpson decrease. Its implementation of new clustering algorithms (SortMeRNA and SUMACLUST) is known to reduce richness overestimation, hence its better stability on varying throughputs. QIIME SS is also the only pipeline where diversity decreases slightly when throughput is increased.

**Table 2:** Comparison of the richness (Chao1) and diversity (Inverse Simpson) indexes for clustering-first pipelines before taxonomic merging, on the 200(V3) HC dataset with sequencing errors simulation when generating 25k, 50k and 100k sequences.

	Chao1			Inverse Simpson		
	25k	50k	100k	25k	50k	100k
**BMP GG**	1580	2780	4731	234.71	278.83	294.1
**mothur S**	1395	2655	4662	118.60	125.67	130.35
**QIIME U GG**	1140	1780	2764	149.27	151.73	154.11
**QIIME SS GG**	696	885	1256	130.31	129.33	129.09

Setting a threshold for read clustering is controversial, since it does not reflect a biological reality: different bacterial taxa can require different cut-off values as they can evolve at variable rates [33]. Using an identity threshold can be even more misleading for error-prone technologies like Ion Torrent, where clustering may be guided more by errors than by taxonomic similarities. The results presented in this subsection show that all pipelines overestimate the diversity and especially the richness indexes. This overestimation is intensified with an increase in the sequencing error rate and throughput. New algorithm developments tend to reduce this effect (for example, the new versions of QIIME), but users still have to keep this phenomenon in mind.

Finally, the F-measure is not significantly impacted by throughput variations. Even though higher throughput generates more small erroneous taxa, causing a richness estimation increase, those taxa concern a small proportion of reads that have a negligible impact on the F-measure calculation. In our context, the pipelines are impacted by read throughput, but the sample complexity can also impact their performance.

### With error-prone reads, all pipelines tend to overestimate the diversity complexity

Bacterial proportions of different microbiomes can vary according to their nature. Table 3 highlights the impact of the bacterial composition and distribution of microbiomes on the richness estimation for datasets of varying complexities, with the three levels LC, MC and HC. (LC contains 30% of one species, MC contains 4 main species at 20% of reads for each one, and HC contains all genomes in similar proportions.) As observed previously on Fig 4 with the HC dataset, all pipelines tend to overestimate the richness on error-prone reads. This is confirmed with the LC and MC datasets. It is worth noting that the number of families is closer to ground truth on the MC dataset, where the large majority of reads is contained in three main families. This tendency is emphasized on clustering-first datasets before taxonomic merging, where richness is overestimated two to three times more on the HC dataset than on the MC one. A possible explanation is that datasets where the majority of reads are in small taxa, such as HC and LC, are harder to grasp. Complexity variation does not impact the richness estimation of error-free amplicons significantly.

**Table 3:** Chao1 values before taxonomic merging for clustering-first pipelines, and at the family level after taxonomic merging for all pipelines, at three different complexities, on the 50k 200(V3) with error simulation datasets. LC, MC and HC were all composed of 50 bacterial families, in varying proportions.

	Before taxonomic merging			After taxonomic merging (families)		
	LC	MC	HC	LC	MC	HC
**BMP GG**	2184	1066	2780	67	66	73
**CLARK**				73	64	78
**Kraken**				53	49	54
**mothur S**	2111	1167	2655	54	52	54
**One Codex OC**				72	58	76
**QIIME U GG**	1370	713	1780	69	66	72
**QIIME SS GG**	773	422	885	67	60	68

**Fig 6:**
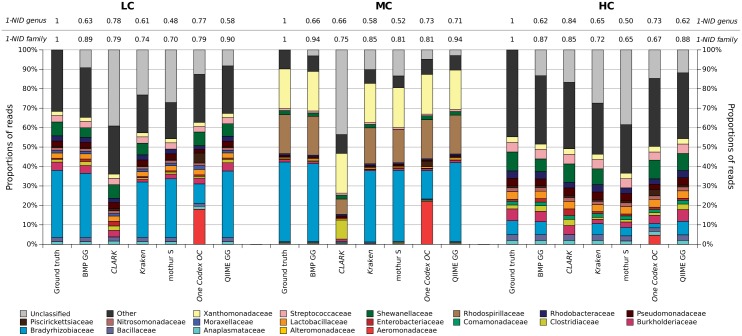
Proportions of the top 10 families per pipeline on the LC, MC and HC 50k 200(V3) with error simulation datasets, and their matching 1-NID clustering indexes (computed after taxonomic merging) at the genus and family levels.

The proportion of the top 10 families after taxonomic assignment is represented in Fig 6, for all pipelines dealing with the three levels of complexity LC, MC and HC. The 1-NID clustering index is used to evaluate how estimated bacterial proportions are fitting the ground truth. Even if a pipeline has a low F-measure due to many false positives, a high 1-NID value indicates that it stills separates the taxa into correct proportions. Clustering index values at the genus level are harder to interpret than at the family level, because of the coarser taxonomic resolution, which all pipelines are not able to reach. Low 1-NID values at the genus level are usually caused by many unclassified reads.

Regarding the LC dataset, all clustering-first pipelines are able to recover the dominant family, Bradyrhizobiaceae family (in blue), corresponding to the dominant species. This is not the case for assignment-first pipelines. One Codex generates a surprising family profile, because many Bradyrhizobiaceae reads are mislabeled as Aeromonadaceae (in red), the latter being more represented in the One Codex 28k database. However, it is the pipeline the closest to ground truth at the genus level in terms of proportions, according to its 1-NID value. Its lower false negative rate at this resolution counterbalances the wrong proportions. CLARK completely fails to identify the Bradyrhizobiaceae family, and is unable to annotate the corresponding reads because they share too many k-mers with other families. These observations also apply to the MC dataset, consisting of four dominant species gathered in three families. All pipelines correctly recognize two out of the three families (Xanthomonadaceae and Rhodospirillaceae). Once again, One Codex and CLARK misclassify the Bradyrhizobiaceae family, which is represented by two dominant species in this sample. CLARK mislabels Rhodospirillaceae reads as Clostridiaceae.

Overall, the clustering-first pipelines closest to ground truth at the family level are QIIME and BMP, properly identifying major taxa with close 1-NID values (>0.86). They are already the best-performing pipelines for the HC datasets, which are the most complex ones, and are therefore able to properly retrieve proportions of lower complexity datasets. Mothur is further from ground truth for all datasets, basically because it has one of the biggest unclassified read rates (LC~27%, MC~13%, HC~38%). It does also not recover the Shewanellaceae family with the SILVA database, being only able to identify the Alteromonadales order at best for those reads.

Those observations are validated on the 25k and 100k datasets and with the other clustering indexes (S1 File). All pipelines have more trouble delimiting a lot of small taxa in a very heterogeneous metagenome (HC) than a more homogeneous one composed of few major taxa (MC), except for CLARK that shows great results on the HC dataset, especially at the genus level (1-NID = 0.86). Complexity especially impacts the richness estimation, in a dramatic way when computed before taxonomic merging for clustering-first pipelines. Those results reveal that a low complexity sample does not mean an easier analysis: The low complexity dataset is more complex to analyze than the medium complexity one, because it contains a higher abundance of small taxa, hence lower 1-NID family values for most pipelines.

### Changing the database has a higher impact on richness estimation than on F-measure

Whether for clustering-first or assignment-first pipelines, one key component of any taxonomic targeted metagenomics analysis is choosing the reference database. This database must be accurately aligned, well annotated, as exhaustive as possible and should also have a normative taxonomy. The simulated datasets of this study were not designed to estimate database content variations: the selected genomes are sufficiently well described in all databases not to interfere with the precision and recall calculations. However, all databases are not built and integrated in the same way into all pipelines; therefore, a change of the database still has an impact on a pipeline's results. Each pipeline advises a default database: for instance, mothur recommends read alignment using SILVA 119 and classification using the RDP v. 9 taxonomy, whereas QIIME and BMP prefer the Greengenes 13.8 database and taxonomy. Databases used with clustering-first pipelines can also limit the taxonomic resolution that can be reached. For example, mothur does not include taxonomic annotations at the species level. For the QIIME integrated databases, less than 7% of Greengenes sequences and less than 45% of SILVA sequences are annotated at the species level.

The authors of Kraken propose an alternate smaller database to reduce computing requirements, named MiniKraken, containing 10,000 k-mers selected from the original RefSeq database. We decided to use MiniKraken in this study as it would probably be the choice of most users. One Codex's results were generated on both RefSeq 65 and the One Codex 28k (displayed by default) databases. The latter includes the RefSeq 65 database as well as 22,710 additional genomes from the NCBI repository. One Codex also beta-implemented a version of the SILVA 119 database. Finally, CLARK provides the binary for the users to build their k-mer database at a specific taxonomic level by extracting bacteria, viruses, human and/or custom genomes from RefSeq at this level (in our case, genus bacteria). The database generated has the particularity of containing k-mers specific to each taxon only.

**Fig 7:**
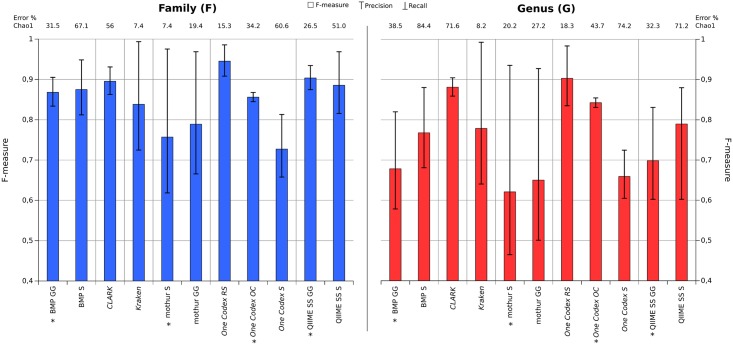
F-measure and richness index error percentage after taxonomic merging for each pipeline on the 200(V3) 50k HC dataset with error simulation, when using different databases (the recommended database for each pipeline is marked with *).

The impact of the database on the pipelines' results can be seen on Fig 7. For clustering-first pipelines, the F-measure is not significantly impacted. However, the richness estimation varies greatly, especially for BMP and QIIME. For these pipelines, using SILVA instead of Greengenes at least doubles the richness estimation at the family level, even if precision is improved. Mothur is more robust when changing the database, mainly because of its lower recall rates.

Kraken shows the smallest Chao1 error percent as well as the best precision. It also exhibits a lower recall, which could come from the small number of k-mers present in the MiniKrakenDB. One hypothesis is that this could be improved using the complete RefSeq database if memory resources allow it. CLARK, which uses a complete RefSeq database as well as and a more precise taxonomic assignment algorithm, has indeed a better F-measure (caused by a better recall) than Kraken. However, its results also contain more noise, with the biggest Chao1 error % caused by small wrongly identified taxa, and hence a lower precision than Kraken.

For One Codex, surprisingly, using the advised One Codex 28k database does not improve the results over RefSeq, in fact quite the opposite: the Chao1 error % is bigger and the F-measure F and G is lower. This could indicate that the added reference sequences, not validated in RefSeq, cause a drop in precision, k-mers matching sequences more diverse in taxonomy. The worse results are obtained with the SILVA database, mainly because it has not been properly curated. A lot of reference sequences are annotated as "environmental" or "uncultured". Reads matching those sequences are either considered separate taxa, or assigned at a much coarser taxonomy, even root, hence resulting in a worse richness estimation and F-measure.

Richness overestimation is a well-known problem for targeted metagenomics analyses on error-prone technologies [34]. Clustering-first pipelines all advise the use of the database minimizing this bias, while assignment-first pipelines are heavily sensitive in the annotation of reference sequences to estimate an exact number of taxa.

### On a real dataset, pipelines also segregate according to their algorithmic approach

**Fig 8:**
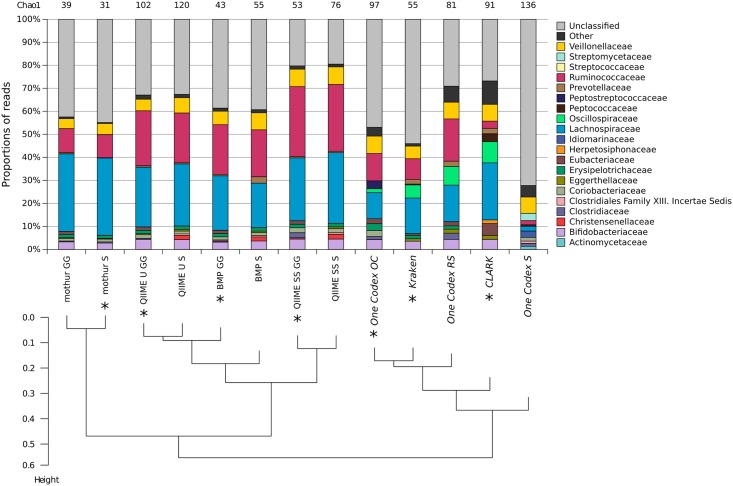
Proportions of the top 10 families per pipeline on a real dataset, and their matching Chao1 diversity indexes (computed after taxonomic merging) at the family level. Below, average linkage hierarchical clustering of all pipelines based on a Euclidean distance calculation on the amount on all reads per family per pipeline (excluding unclassified reads). Pipelines are marked with a * when executed with their default database.

Contrary to simulated data, real datasets can contain some noise which cannot be simulated. In order to validate the main conclusions drawn with simulated data, a real dataset was analyzed with the 6 pipelines. The considered sample (SRX364048 on SRA) contained 231,660 reads on the 200(V3) amplicon, using the same primers as our simulated data. This real dataset contains more noise (mean: 151 nt, standard deviation: 45 nt) and a global lower quality than the 200(V3) 100k simulated dataset (mean: 185 nt, standard deviation: 11 nt). Fig 8 represents the taxonomic assignments obtained with the different pipelines on this dataset, with varying databases. The first observation is the high amount of unclassified reads for all pipelines. This can be caused by the experimental noise, as well as a richer microbiome with some organisms less well described in the different databases. The original article from which this dataset was selected used QIIME UCLUST for taxonomic content analysis. They described three major families (Lachnospiraceae, Ruminococcaceae and Veillonellaceae) on the sample group this dataset comes from. Those three families are recovered with all pipelines, except One Codex SILVA which has the worst performance. Oscillospiraceae is a family only retrieved by assignment-first pipelines based on nonribosomal specific databases. The same reads are identified as Ruminococcaceae for the other pipelines.

All pipelines' results are clustered in Fig 8 based on the proportions of families they are able to recover. Clustering-first pipelines are all grouped on the first branch of the hierarchical clustering tree. QIIME U, QIIME SS and BMP are clustered together since they are based on similar algorithmic steps and use the same taxonomic classification algorithm. QIIME SS is the most up-to-date clustering-first pipeline, lowering the amount of unclassified reads and the richness estimation. It is the one of the best-performing pipelines on our simulated datasets. Mothur is also grouped with the clustering-first pipelines, but has a higher amount of unclassified reads, which fits with its low recall values observed in simulated datasets—and explains its low estimation of richness.

A beta-diversity analysis has been performed on the real dataset results of all pipelines, assuming they can be considered as different biodiversity contexts. The resulting Principal Coordinates Analysis (S2 File), based on a Bray-Curtis distance matrix, confirms the pooling of the pipelines previously observed with the hierarchical clustering Fig 8.

For clustering-first pipelines, lower richness is retrieved when using the default database, as shown in the previous section studying the impact of databases on the simulated datasets. The real dataset helps to reveal another phenomenon relating to databases: the importance of the divergent taxonomies. For example, all pipelines using Greengenes identify a proportion of reads from the *Eubacterium* genus classified as Eubacteriaceae, which seem absent when using SILVA, where they are in fact classified under the Erysipelotrichaceae family. A good knowledge of the taxonomy of the database used in a targeted metagenomics study is therefore crucial for results interpretation.

Assignment-first pipelines are all grouped together by the hierarchical clustering. CLARK is the assignment-first pipeline able to identify the highest amount of reads, especially at the genus level (S1 File). One Codex and Kraken, having the same k-mer alignment + LCA algorithmic approach, have similar behaviors. One Codex OC is not able to identify many reads (unclassified) because of the One Codex 28k database, as shown in the previous section. Using One Codex with SILVA results in a huge amount of unclassified reads (>60%) because of the high number of unclassified sequences in the database. Only One Codex RS is able to decrease the unclassified rate because of the RefSeq database which is well annotated and validated. Kraken displays many unclassified reads, which are largely identified at coarser resolutions of taxonomy because of using a reduced database (MiniKraken). This real dataset was re-analyzed with Kraken and the complete RefSeq database and did indeed generate close results to One Codex RS, proving once again the importance of exhaustive and well-annotated databases for assignment-first methods.

A Mann-Whitney-Wilcoxon test was performed to compare the estimated richness and diversity indexes between both groups, clustering-first and assignment-first pipelines, already segregated by the hierarchical clustering. The estimated diversity was significantly higher for assignment-first pipelines (p < 0.005 for Shannon and Inverse Simpson index), validating the segregation of clustering-first pipelines and assignment-first pipelines. However, no significant difference was noted for richness estimation between the two groups, probably because of the highest sensitivity of this index to sequencing noise which is more present in real datasets, and handled quite differently depending on the pipelines, as shown in the previous sections.

### Some standalone pipelines can be computationally demanding regardless of results quality

In addition to sample preparation and sequencing, runtime and memory requirements are important factors to consider in targeted metagenomics studies and should not be underestimated. QIIME, BMP, mothur, CLARK and Kraken were evaluated on their memory requirement, with the RAM usage, and running time with the CPU time and the total execution wall time. We were not able to perform this evaluation for One Codex, which is not a standalone program and is only accessible through a website.

**Fig 9:**
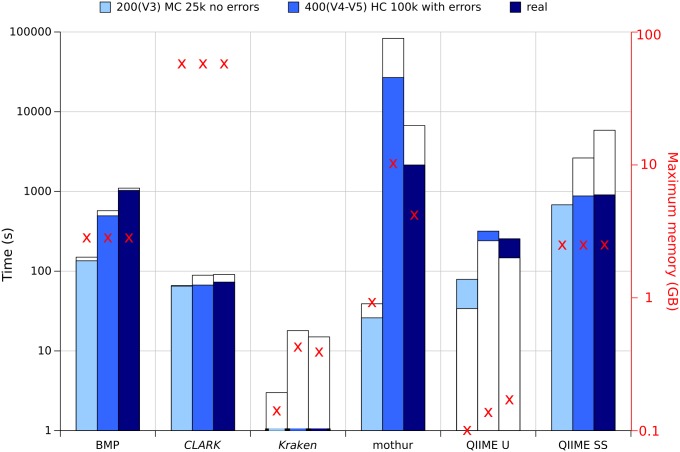
Histogram of wall time (colored) and CPU time (white), and peak memory usage (red crosses) for each standalone pipeline on three different datasets.

Fig 9 shows the peak memory usage of each pipeline for three different datasets, as well as the wall time and CPU time required to analyze them.

Kraken is by far the fastest pipeline and one of the most memory-efficient ones, using below 500MB of memory for all datasets and requiring only 1 second, thanks to the reduced MiniKraken database. By default, the database is not preloaded into memory. Such preloading using a RAMDisk is possible and decreases Kraken's execution time, but requires RAM space at least equivalent to the size of the database. This tradeoff should be considered when using the complete RefSeq database which might increase running times considerably.

CLARK is supposedly faster and less memory-intensive than Kraken [21], mostly because its database only contains discriminant k-mers at a specific taxonomic level. However, as shown in Fig 9, using a RefSeq k-mer database (even if it contains anly discriminant k-mers) is much more resource-intensive (up to 160 GB RAM) than the subsampled database approach used by Kraken with MiniKraken (<1 GB RAM). A smaller k-mer database maintains the precision of results at a lower memory overhead, but as explained beforehand, a complete k-mer database allows better recall. CLARK proposes an alternative, less memory-intensive algorithm (CLARK-l) which has not been tested in this study. Like Kraken, CLARK also optionally allows the database to be loaded into memory, which lowers the running time but increases memory consumption.

QIIME U is the most memory-efficient pipeline, using at most below 150MB, with a very low variation between datasets. It is also the fastest clustering-first pipeline, running under 5 minutes for the real sample. Surprisingly, its parallelized clustering step requires more wall time than CPU time, which is perhaps caused by a results merging step. BMP's clustering step does not require the alignment of the reads with a reference database; therefore, it should be quite fast. However, the pipeline includes a phylogenetic tree generation step, which requires a read alignment to the reference. This step lengthens execution times, and can be optional if not needed by the user. QIIME U and BMP both use UCLUST, for which the free version is 32-bit compiled and has a maximum of 4 GB RAM usage limitation (2 GB in Windows). This limit has not been reached in this study, but could be a limiting factor when using bigger databases. The 64-bit binary lifts this limitation, but requires a paid license. The QIIME development team looks to make QIIME fully open-source, hence the latest implementation of recent open clustering algorithms like SortMeRna, Sumaclust and SWARM in the 1.9 version in QIIME SS. QIIME SS requires more memory and time than its predecessor, as the OTU picking step is more time- and memory-intensive (around 2.5 GB for all datasets) but with better results.

Mothur is the most variable pipeline in this study, and this appears through computing resources consumption. The biggest memory bottleneck is the splitting of the distance matrix file, from 1.4 GB for LC 25k on 200(V3) to 45.5 GB for MC 100k on 400(V4-V5); the bigger the matrix, the more memory is required to split it, and also the more wall time and CPU time. For the latter dataset, wall time reaches more than 7 hours. Disk space must also be kept in mind for this pipeline, using up to 16.1 GB of disk space for the 400(V4-V5) HC 100k dataset with error simulation, because of the huge distance matrix file.

Web servers such as One Codex spare the user from computing resources considerations, but nonetheless have their own limitations. First of all, they require a decent and stable Internet bandwidth, since all raw sequencing files must be uploaded. For this study, all the raw datasets add up to 1.62 GB that have to be transferred to external servers before being analyzed. One Codex allows a maximum of 5 simultaneous uploads, and files up to 5 GB maximum, which could be limiting the uploading process. One Codex analyzed all datasets in seconds, making it as impressively performing as other assignment-first pipelines in terms of execution times. However, it requires more human time to upload and retrieve files, even if an API is under development. For this pipeline, the users are dependent on external computing resources over which they have no control.

## Discussion

Computational approaches to analyze targeted metagenomics data have been developed in parallel with the popularization of this new application. Historically, the first tools like DOTUR (Schloss, 2005) clustered sequences into OTUs based on the genetic distances between sequences. The subsequent tools dedicated to targeted metagenomics analyses have been built on this approach and have continuously improved it. Currently, all popular pipelines used in a targeted metagenomics context are based on the clustering-first approach. One can consider three main criteria that make a pipeline popular: its historical seniority, the number of published works in which it is used, and finally its user-friendliness. Indeed, an accessible and intuitive pipeline has the opportunity to become widely used, beyond its performance. On the contrary, standalone command-line pipelines requiring bioinformatics skills may be an obstacle for users not familiar with command-line tools. Using a web-based interface simplifies the analyses by reducing the number of available options and parameters, and also outsources all computing calculations. The main disadvantages of those solutions are their lack of transparency and customizability, as well as the increasing queuing delay for widely used pipelines. They are, however, helpful for people with limited computing resources, as all calculations are outsourced.

Computing resources could be limiting when analyzing whole WGS metagenomic datasets, for which the use of clustering-first algorithms is not appropriated, hence the development of innovative algorithmic approaches different from OTU clustering to analyze this data. These assignment-first approaches are recent and can be considered as emerging in the context of targeted metagenomics. Indeed, these methods were not developed for targeted metagenomics and are not used in this context yet, even if they may be effective and promising.

In this paper, we presented an evaluation of pipelines in the Ion Torrent 16S rDNA sequencing context, introducing a complete evaluation protocol with simulated and real datasets and adapted metrics.

**Fig 10:**
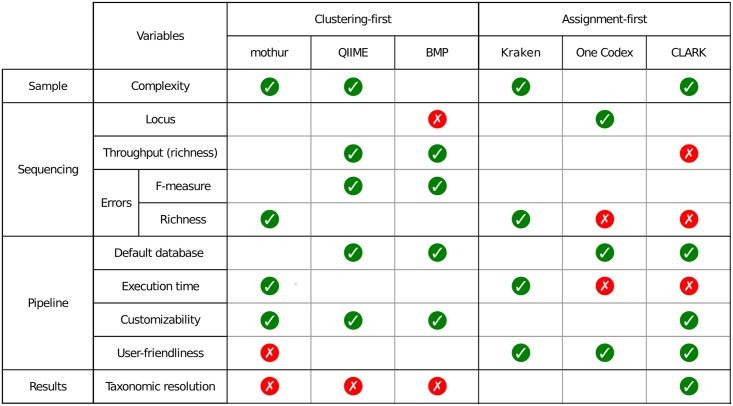
Performance summary of each pipeline (default databases) when varying different parameters. Colored disks represent how each pipeline handles specific variables (red cross = bad, green check = good, no disk = no major impact).

Six pipelines were compared: three popular clustering-first pipelines and three emerging assignment-first pipelines. We showed that all pipelines are able to identify the coarse bacterial profile of real and simulated samples, but they deliver significantly different results. Fig 10 summarizes the pipelines' behaviors and the fact that they are not all sensitive to the same variables. Fig 10 also reveals how the pipelines were discriminated according to their algorithmic approach: assignment-first pipelines are able to reach coarser taxonomic resolutions, whereas clustering-first tools are more robust for richness evaluation under various throughputs and when errors are present.

Concerning clustering-first pipelines, mothur seems less effective in our study context, probably because it was developed under the assumption of high-quality sequencing results, and is therefore not well fitted for error-prone technologies like Ion Torrent sequencing. BMP guidelines have considered these Ion Torrent technological specificities but the produced results are not the best among the clustering-first pipelines. Better performance is achieved with the integration of recent algorithms like SortMeRna and Sumaclust in QIIME [35]. These new algorithms reduce the well-known richness overestimation problem of OTU-based approaches, when richness is estimated before taxonomic assignment, and is especially important when dealing with error-prone sequences. Error-prone reads reduce the similarity of all sequences belonging to a single taxon, and make a fixed similarity threshold (usually 97%, which is often criticized [36] unsuitable for OTU clustering). New clustering algorithms without a fixed threshold, like SWARM [33], are emerging to counter this bias and are starting to be implemented in pipelines, such as the QIIME *de-novo* clustering pipeline.

Preprocessing steps represent a solution of choice to reduce the sequencing errors and minimize their impact on clustering-first pipelines. Denoising [37,38], error-correction [39] and read trimming processes improve the quality of the reads, and are strongly recommended to reduce the richness overestimation. However, those methods are unfortunately not standardized yet and sometimes difficult to adapt to the Ion torrent data [40–41].

This study also confirmed that assignment-first pipelines could be used in targeted metagenomics studies, in the context of an error-prone technology and with fast execution times. However, these pipelines are highly dependent on the reference database, which is often not suitable for studying a specific locus only, and causes richness overestimation. Comparing the use of a complete RefSeq k-mer database with CLARK and a subsampled k-mer database (MiniKraken) with Kraken revealed that reducing the size of the k-mer databases reduces the required computing resources with no significant drop in precision values, but decreases recall values. The results also highlighted that the algorithmic improvements of the taxonomic assignment steps in CLARK allow a finer taxonomic resolution to be achieved. The recent development of algorithms using spaced k-mers seems promising [42], and their implementation into new pipelines could improve those results still further. Finally, the adaptation of these emerging approaches to targeted metagenomics analyses requires adequate well-annotated databases that have yet to be developed. It is worth mentioning that, in spite of their promising results and performance, these pipelines are severely limited when studying an exotic microbiome that is not well described in the databases, and for which clustering-first approaches are more suited to identifying taxonomic units.

In order to perform a proper evaluation of pipelines dealing with targeted metagenomics data, this article introduced an evaluation protocol for targeted metagenomics analysis pipelines that could be used on other genomic targets and sequencing technologies. Simulated datasets and fitted metrics allow the precise evaluation of the closeness of any pipeline's results to ground truth, regarding the richness estimation and taxonomic identification. These datasets are openly available on http://www.pegase-biosciences.com/metagenetics/ and can be used by pipeline developers and users to help them refine their parameters. They could also be used as monitoring datasets to add a critical perspective on the analytical processes used in any targeted metagenomics study. It should always be kept in mind that the final results of a targeted metagenomics analysis are dependent on the experimental design, the analytical pipeline, and the related database and parameter profiling: newer bioanalytical approaches and technical improvements can lead to different results, which may lead to different biological interpretations.

## Materials & Methods

### Human gut microbiome dataset

This dataset was selected from a 16S rDNA human gut microbiome study [23], using Ion Torrent PGM sequencing. It is archived on SRA under the ID SRX364048, and contains 231,660 reads. Its amplification target is the V3 16S rDNA region (~200 bp). The raw sequencing data was trimmed using cutadapt (Martin, 2011), removing the adaptors and sample index.

### Synthetic datasets

Simulated datasets were generated using the FAMeS guidelines adapted by Pignatelli & Moya [31], creating artificial metagenomes containing 125 distinct bacterial genomes (51 families and 69 genera) distributed into three different complexities (LC, MC and HC) defined as follows:

- LC, Low Complexity: 30% of *Rhodopseudomonas palustris HaA2* (NC_007778.1), which belongs to the family Bradyrhizobiaceae. Other organisms equally distributed.
- MC, Medium Complexity: 20% of *Rhodopseudomonas palustris HaA2* (NC_007778.1), *Rhodospirillum rubrum ATCC 11170* (NC_007643.1, family Rhodospirillaceae), *Bradyrhizobium sp*. *BTAi1* (NC_009485.1, family Bradyrhizobiaceae) and *Xylella fastidiosa M12* (NC_010513.1, family Xanthomonadaceae). All other organisms are equally distributed. Note that the MC datasets contain four dominant species of different genera, but represent only three families.
- HC, High Complexity: no dominant organism, all equally distributed.

Targeted metagenomics reads were simulated using two different 16S rDNA amplicons, popularly used for bacterial studies:

- 200(V3) is the amplicon generated by Probio_Uni and Probio_Rev primers [23], surrounding the 16S rDNA V3 region (~200 bp).
- 400(V4-V5) is the amplicon generated by the 519F and 907R primers (Lane, 1991; Stubner, 2002), surrounding the 16S rDNA V4-V5 regions (~400 bp).

Those amplicons were selected based on two criteria: their primers should avoid amplifying nonbacterial organisms like contaminants or organelles [43], and should surround hypervariable regions as discriminant as possible between bacterial taxa [44]. Those two pairs of primers cover 82.1% and 83% of the bacterial domain for 200(V3) and 400(V4-V5) respectively, according to TestPrime (Klindworth, 2012) on the SILVA SSU r122 database.

Grinder [45] was used to extract amplicons from complete genomic sequences. This software fetches amplicons randomly across the several potential 16S rDNA copies within a genome sequence. Simulated amplifications were performed without allowing any primer mismatches on the 125 genomes listed by Pignatelli & Moya; therefore, 15 of the 125 genomes were not amplified with either of the two pairs of amplicons. Two species were only amplified with the 400(V4-V5) primers: *Leuconostoc mesenteroides* and *Thermobifida fusca*, finally leading to 104 amplicons for the 400(V4-V5) primers, and 102 amplicon sequences for the 200(V3) primers. We used Grinder at three different throughputs: 25,000, 50,000 and 100,000 amplicons were generated for each of the three complexity levels. Moreover, for each sequencing throughput and complexity, we constructed two datasets. The first one is composed of full-length error-free amplicons, with no sequencing errors. The second has error-prone reads with simulated sequencing errors and read lengths. For the latter, CuReSim [11] was applied to simulate an Ion Torrent read size and error model (0.01 deletion rate, 0.005 insertion rate, 0.005 substitution rate, and a 20 bp standard deviation from the amplicon size).

### Pipeline settings

Standalone pipelines (mothur, QIIME, BMP, Kraken and CLARK) were executed on the same hardware, with 2 Intel Xeon E5-2470 CPUs and 192 GB RAM. All steps were parallelized when possible using 32 cores. Preprocessing guidelines were adjusted to input datasets: sequence trimming steps were turned off, except for BMP which requires a fixed sequence size. Chimera detection was also turned off, since no chimeras were simulated and all pipelines did not include a chimera removal step. Taxonomic singletons (taxa containing only one read) were discarded from the final results and considered false negatives. Indeed, clustering-first pipelines discard singletons in their guidelines (QIIME has a minimum OTU size default value of 2, BMP discards singletons and Mothur has a normalizing step which *“will remove a few groups that that didn't have enough sequences”*). We removed singletons in assignment-first pipelines results to match those guidelines, since there are none for using assignment-first pipelines on targeted metagenomics datasets yet.

Each pipeline was executed to be as well fitted as possible to Ion Torrent data. Mothur (version 1.35.1) provides guidelines for 454 and Ion Torrent, which only differ in the read trimming step which was turned off in our context. QIIME (version 1.9.0) was used with the pick_open_reference_otus.py script using the recent open-source integrations of SortMeRNA (Kopylova, 2012) and Sumaclust (Mercier, 2013). The default UCLUST approach has also been executed and significant differences between both approaches are mentioned in the text (the first version being abbreviated QIIME SS, and the UCLUST version being abbreviated QIIME U). BMP (version of Dec. 2014) is the only pipeline proposing specific guidelines for 16S rDNA targeted metagenomics Ion Torrent analysis, using both UCLUST and some steps of QIIME. All clustering-first pipelines were executed on the SILVA 119 database (S, default for mothur), and the Greengenes 13.8 database (GG, default for QIIME and BMP). Assignment-first pipelines were run with their default settings: Kraken (version 0.10.5-beta) using the MiniKraken database (version 20141208, subset of 10,000 k-mers selected from RefSeq), CLARK (version 1.1.2) using the discriminating k-mers bacterial domain of the RefSeq database at the genus level, and One Codex (a proprietary web service using RefSeq (RS), their own proprietary database One Codex 28k (OC) and a beta implementation of SILVA 119 (S)).

### Availability of the evaluation protocol

All raw simulated datasets and a spreadsheet describing the composition of each dataset in details (genome IDs, amplicon positions, read quantity per amplicon, etc.), as well as specific command lines, guidelines and algorithmic approaches used for each pipeline can be found on the following website: http://www.pegase-biosciences.com/metagenetics/

### Taxonomic identification standardization

To compare assigned taxonomies across pipelines and databases, all taxonomic identifications were converted to the NCBI Taxonomy format: E-utilities [46] and homemade Perl scripts were used to retrieve the NCBI taxid of each assigned read based on its annotation at its lowest taxonomic rank, and matching parent taxonomic ranks at the family and genus level.

### Metrics

Three kinds of metrics were used to evaluate the results of each pipeline: F-measure, clustering indexes, and diversity indexes.

In the output of each analysis pipeline on simulated datasets, a read is considered a true positive (TP) at a specific taxonomic level (family or genus) if its taxonomic assignment at this level is the same as the genome it was extracted from. A read is considered a false positive (FP) if this assignment is different. A read is considered a false negative (FN) if it is discarded by the pipeline (e.g. too many homopolymers, bad alignment, etc.) or annotated as “unclassified”. Precision, recall and F-measure are computed as follows:

$$\text{precision}=\frac{\text{TP}}{\text{TP}+\text{FP}}$$

$$\text{recall}=\;\frac{\text{TP}}{\text{TP}+\text{FN}}$$

$$\text{F-measure}=2\times \frac{\text{precision}\times \text{recall}}{\text{precision}+\text{recall}}$$

Clustering and diversity metrics were selected based on previous work [47]. Simulated datasets allowed the comparison of the partitioning similarity between pipelines' results and the ground truth. Clustering metrics (NMI and AMI) were computed between taxa and ground truth at the family and genus level: the closer to 1, the closer the partitioning is to ground truth. The NID (Normalized Information Distance) was also computed [48]. To be concordant with the F-measure and other clustering indexes (the closer to 1, the closer to ground truth), the 1-NID value has been used in this paper. A diversity index (Inverse Simpson) and an abundance-based richness estimator (Chao1) were also computed. Clustering, diversity and richness indexes were calculated on OTUs immediately after OTU clustering for clustering-first pipelines. To compare their performance with assignment-first pipelines, they were also computed after a taxonomic-merging step: all OTUs assigned to the same taxon were merged into a single taxonomic unit. After taxonomic merging, to evaluate how close Chao1 estimations were from ground truth for simulated data at a taxonomic level, Chao1 error percent was computed as follows:

$$\text{Chao1 error percent}=\frac{\text{estimated Chao1}-\text{ground truth Chao1}}{\text{ground truth Chao1}}\times 100$$

Execution time, CPU time and maximum RAM usage were measured for each standalone pipeline step with the UNIX /usr/bin/time command.

### Hierarchical clustering of all pipelines

In the subsection “On a real dataset, pipelines also segregate according to their algorithmic approach” of the Results & Discussion section, all pipelines were clustered using R version 3.0.2 with an average linkage hierarchical clustering, based on a Euclidean distance matrix computation between all pipelines' results for the real dataset. The results consisted of the relative abundance of each family discovered by each pipeline (excluding unclassified reads).

## Supporting Information

S1 File Excel sheets of all metrics for all pipelines (richness, diversity and clustering indices before and after taxonomic assignments, precision, recall and F-measure). (XLSX)

S2 File PcoA analysis using Bray-Curtis distances between pipelines on the real dataset at the family level. (TIF)
