# Supplementary figures and images for: Assessment of Common and Emerging Bioinformatics Pipelines for Targeted Metagenomics
Source: PLoS One. 2017 Jan 4;12(1):e0169563. doi: 10.1371/journal.pone.0169563 (PMC5215245; doi:10.1371/journal.pone.0169563)

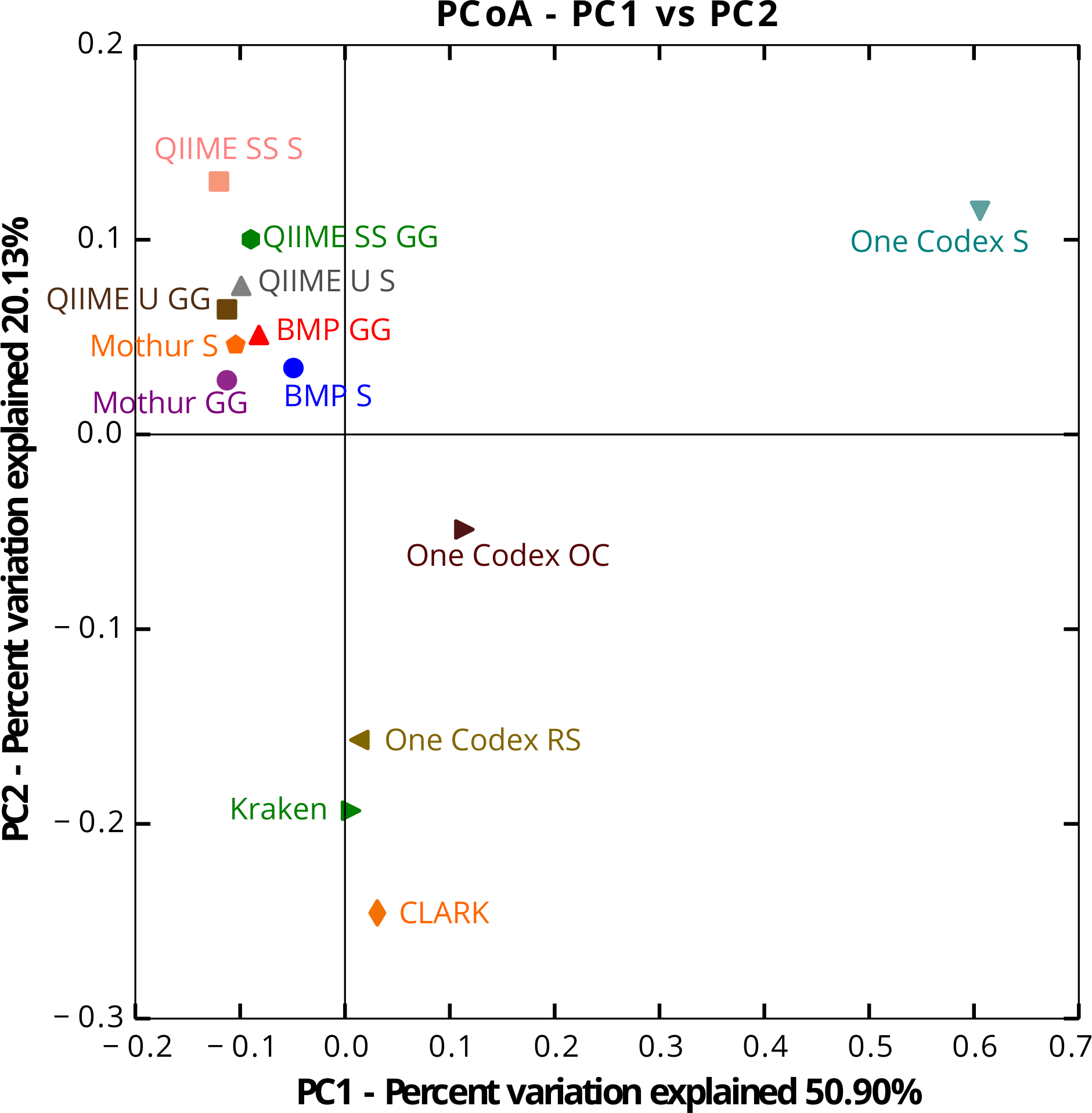

Supplement: S2 File — (TIF) [file pone.0169563.s002.tif]
